# Enhanced photocatalytic performance of a Ti-based metal-organic framework for hydrogen production: Hybridization with ZnCr-LDH nanosheets

**DOI:** 10.1038/s41598-019-44008-6

**Published:** 2019-05-20

**Authors:** Muhammad Sohail, Hyunuk Kim, Tae Woo Kim

**Affiliations:** 1 Energy Materials Laboratory, Korea Institute of Energy Research, 152 Gajeong-ro, Yuseong-gu, Daejeon 34129 Republic of Korea; 2 Advanced Energy Technology, University of Science and Technology, 217 Gajeong-ro, Yuseong-gu, Daejeon 34113 Republic of Korea

**Keywords:** Artificial photosynthesis, Metal-organic frameworks, Photocatalysis

## Abstract

Novel hybrid composites of NH_2_-MIL-125(Ti) and ZnCr-layered double hydroxide nanosheets (ZnCr-LDH NSs) are developed for use as visible-light-active photocatalysts for hydrogen production based on water photolysis. The hybrid composites are obtained by growing NH_2_-MIL-125(Ti) in the presence of exfoliated ZnCr-LDH NSs using a solvothermal reaction. Hybridization of NH_2_-MIL-125(Ti) with exfoliated ZnCr-LDH NSs leads to significant effects on the morphology and optical properties of NH_2_-MIL-125(Ti). To find the optimum photocatalytic activity for hydrogen production by the hybrid composite photocatalysts, the content of ZnCr-LDH in this work is controlled. Compared to that of pristine NH_2_-MIL-125(Ti) and ZnCr-LDH, the hybrid composites exhibit an improved photocatalytic activity for hydrogen production under visible-light irradiation. In addition, the hybrid composite photocatalyst shows excellent photo-chemical stability. The improved photocatalytic activity is believed to benefit from the synergy of strong electronic coupling between NH_2_-MIL-125(Ti) and ZnCr-LDH NSs, expanded light absorption and band alignment to enhance the lifetime of photo-induced electrons and holes.

## Introduction

Over the past decades, the rapid depletion of fossil fuels and the occurrence of environmental pollution have raised awareness to the biggest crisis that humans have ever faced. To overcome it, many efforts have been steadily made in the technological development of alternative clean energy and environmental remediation using sustainable energy sources, such as sun, wind, geothermal power, etc^1^. Among them, visible-light photocatalytic technology using solar energy is very promising for energy and environmental issues due to its abundance, ease of use, and eco-friendliness^1–3^.

Metal-organic frameworks (MOFs), comprised of metal ions and organic ligands, have received significant attention because of their unique structural characteristics including highly ordered and nanoporous networks^4^. Thus, MOFs have numerous potential applications in gas storage, gas separation, sensing, and catalysis^5–8^. Moreover, in recent years, MOFs as photocatalysts for hydrogen (H_2_) production based on water photolysis have received considerable attention^9–14^. Among the various MOF-based photocatalysts, NH_2_-MIL-125(Ti) (Supplementary Information, Fig. S1), which consists of cyclic Ti_8_O_8_(OH)_4_ octamers and 2-aminoterephthalic acid, possesses a narrow band gap of 2.5 eV and has been reported as a potential photocatalyst that is able to produce hydrogen under visible-light irradiation^15–19^. However, due to the inefficient charge transfer, light absorption, and photo-chemical stability, the photoactivity of NH_2_-MIL-125(Ti) towards hydrogen production from water is not very effective in comparison with traditional semiconductor photocatalysts^17–20^. Therefore, it remains an important challenge to improve the photocatalytic performance of NH_2_-MIL-125(Ti) photocatalysts. One of the viable strategies to enhance the photocatalytic activity of NH_2_-MIL-125(Ti) is to hybridize it with other semiconducting photocatalysts; such a hybridization can facilitate electron transfer between two components, and thus, the photocatalytic activity of the MOF can be enhanced^21,22^.

Herein, we report novel NH_2_-MIL-125(Ti)-based hybrid composites that show enhanced visible-light photocatalytic activity towards hydrogen production based on water photolysis. The hybrid composites are prepared by the hybridization of NH_2_-MIL-125(Ti) and layered double hydroxides (LDHs) with a two-dimensional structure. Of course, different composites of MOF/0D-semiconductors and/or MOF/2D-graphene as photocatalysts for water remediation have been reported in recent studies^23,24^. However, a few reports on hydrogen production via water photolysis using MOF-based hybrid composites have been reported^17–20,22^. Very recently, hybrid composites of MOFs and LDHs have been examined as gas and water adsorbents^25,26^. To the best of our knowledge, however, there has been no report on improving photocatalytic hydrogen production through the hybridization of MOFs and LDHs.

In this work, ZnCr-LDH, which is comprised of Zn^2+^ and Cr^3+^ ions, was used. This material has been reported as a very promising photocatalyst with a narrow band gap of 2.6 eV and the appropriate valence band position to oxidize water^27^. At 420 nm, its quantum efficiency is above 60%, and the material exhibits an excellent photo-chemical stability^27^. Therefore, it was believed that the hybridization of NH_2_-MIL-125(Ti) with ZnCr-LDH would result in a positively synergetic effect to improve the photocatalytic efficiency under visible-light irradiation.

Hybrid composites of NH_2_-MIL-125(Ti) and ZnCr-LDH nanosheets (NSs) were synthesized by growing NH_2_-MIL-125(Ti) in the presence of exfoliated ZnCr-LDH NSs, as illustrated in Scheme 1a. It has been reported that LDHs can be prepared as a colloidal suspension of individually exfoliated NSs in a non-aqueous solvent (i.e., formamide)^28–30^. Typically, NH_2_-MIL-125(Ti) is synthesized in the similar solvent such as dimethylformamide (DMF, Scheme 1b)^17,31^. With this understanding of the reaction medium, the nanosheet colloids were first induced from bulk ZnCr-LDH in a mixed non-polar solution (DMF/MeOH, 50:50 wt%).

**Figure 1:**
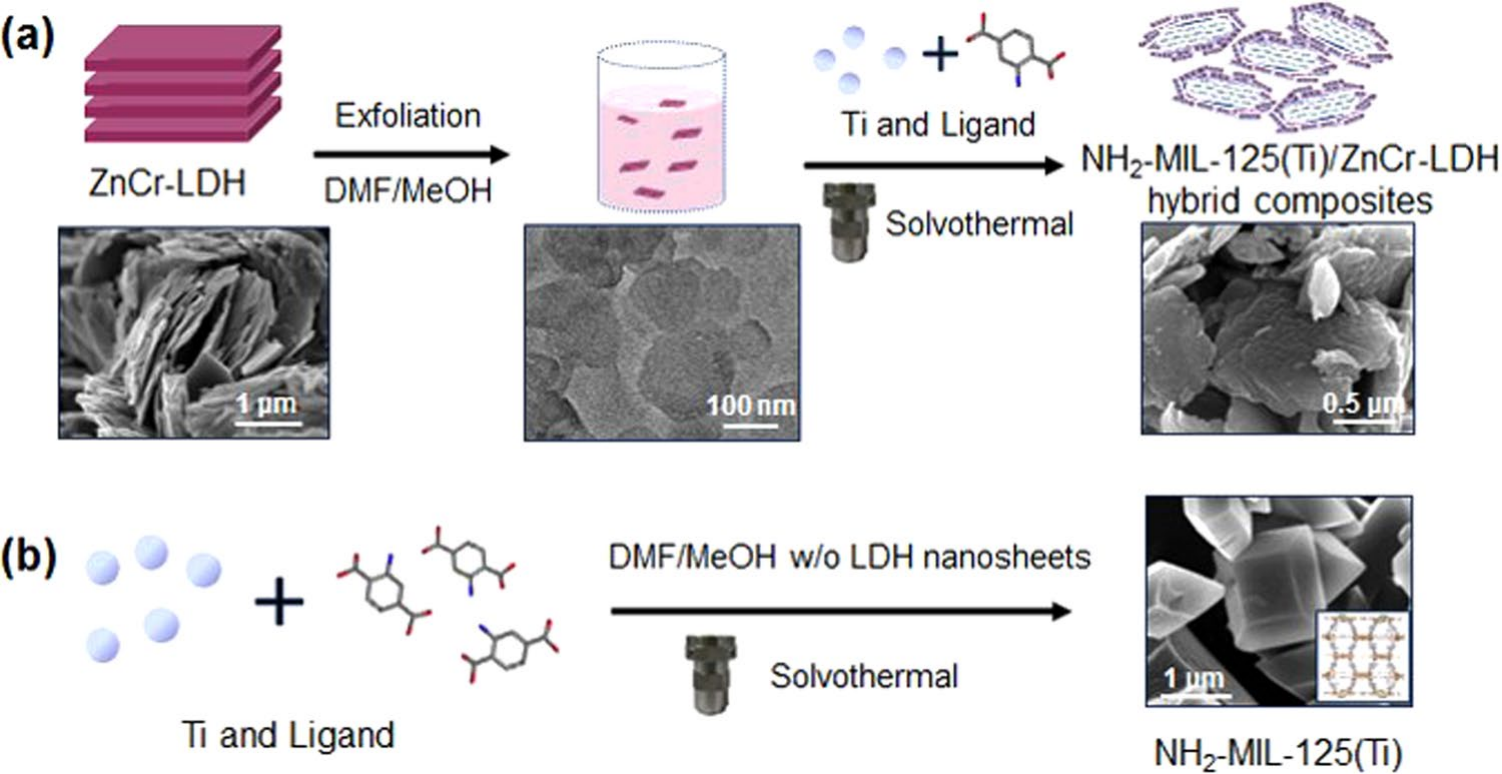
Illustration of the syntheses of (**a**) hybrid composites of NH_2_-MIL-125(Ti) and ZnCr-LDH NSs and of (**b**) pristine NH_2_-MIL-125(Ti). FE-SEM images corresponding to each figure are also shown.

Pristine ZnCr-LDH in its nitrate form was prepared by the co-precipitation method^30^. From powder X-ray diffraction (powder XRD), it was confirmed that the synthesized ZnCr-LDH exhibits typical Bragg reflection patterns of the hexagonal phase ions (Supplementary Information, Fig. S2a). Field-emission scanning electron microscopy (FE-SEM) images reveal a plate-like morphology of submicron size for the pristine ZnCr-LDH, as shown in Fig. 1a. The colloidal suspension of exfoliated ZnCr-LDH NSs was obtained in the mixed solution of DMF/MeOH by alternatively performing ultrasonication and vigorous stirring. The resultant colloidal suspension has a bright-purple colour, and clearly shows the Tyndall phenomenon by laser illumination (Supplementary Information, Fig. S3a). This result is indicative of the presence of tiny particles in the solvent. The Zeta potential measurement of the exfoliated ZnCr-LDH NSs reveals a positive value of approximately 23 mV (Supplementary Information, Fig. S3b). This value indicates that ZnCr-LDH NSs have a positively charged surface, which is in agreement with the previously reported result^30^. For the exfoliated ZnCr-LDH NSs, the transmission electron microscopy (TEM) images show a plate-like morphology with a very thin thickness (Figs 1a, and S4a). Unlike that of the bulk ZnCr-LDH, no powder XRD pattern of the exfoliated ZnCr-LDH NSs is observed, as they typically show an amorphous structure (Supplementary Information, Fig. S2b). This result is due to the loss of long-range stacking in the c-axis^28–30^. However, the selected area electron diffraction (SAED) image clearly shows that the exfoliated ZnCr-LDH NSs present hexagonal spots (Supplementary Information, Fig. S4b). This pattern clearly indicates that the crystal structure of ZnCr-LDH NSs remains undecomposed after the exfoliation reaction^28–30^. The hybrid composites of NH_2_-MIL-125(Ti) and ZnCr-LDH NSs were synthesized by putting the precursors of NH_2_-MIL-125(Ti) into the colloidal suspension of ZnCr-LDH NSs and proceeding with the solvothermal reaction. To find the optimum performance of photocatalytic hydrogen production, a series of hybrid composites were prepared by controlling the weight of ZnCr-LDH powder (50, 100, 200, and 400 mg) used for the colloidal suspension of ZnCr-LDH NSs, and the obtained hybrid composite samples were named as ML50, ML100, ML200, and ML400, respectively.

**Figure 2:**
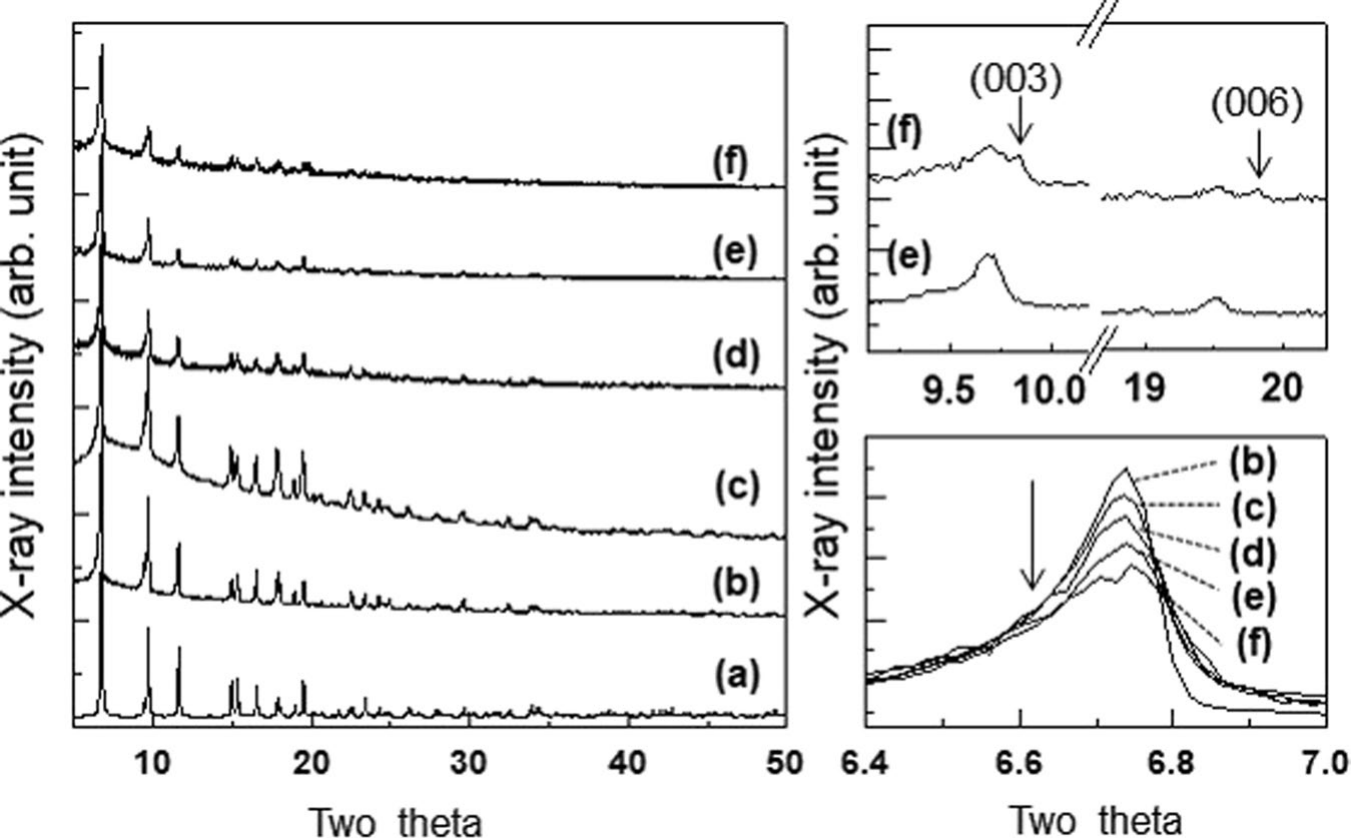
(Left panel) power XRD patterns of (**a**) simulated NH_2_-MIL-125(Ti), (**b**) pristine NH_2_-MIL-125(Ti), (**c**) ML50, (**d**) ML100, (**e**) ML200, and (**f**) ML400. (Right panel-top) The enlarged areas of (**e**,**f**) in the range between 9.1° and 20.3°. (Right panel-bottom) The enlarged area of the main peaks in the range between 6.3° and 7.1°.

The crystal structures and phase purities of the synthesized hybrid composites were examined by powder XRD patterns. As plotted in the left panel of Fig. 2, the powder XRD pattern of pristine NH_2_-MIL-125(Ti) prepared without ZnCr-LDH NSs matches well with the simulated one, and impurity peaks, such as those of TiO_2_, are not observed. After the hybridization with ZnCr-LDH NSs, the powder XRD profiles exhibit only NH_2_-MIL-125(Ti) peaks, regardless of the different amounts of ZnCr-LDH NSs except for ML400. This suggests that ZnCr-LDH NSs are homogeneously distributed in the present composite materials. In the case of ML400, however, reflection peaks of ZnCr-LDH corresponding to the (003) and (006) planes were observed, as shown in the right panel-top of Fig. 2. These impurities are attributed to the fact that a portion of the bulk ZnCr-LDH content used for the preparation of ML400 could not be exfoliated under our synthesis conditions. A close inspection of XRD profiles reveals that the main peak intensity approximately 6.74° becomes suppressed with increases in the amount of ZnCr-LDH, as seen in the right panel-bottom of Fig. 2. This phenomenon may be attributed to the limited crystallization of NH_2_-MIL-125(Ti) by ZnCr-LDH NSs during the formation of NH_2_-MIL-125(Ti).

**Figure 3:**
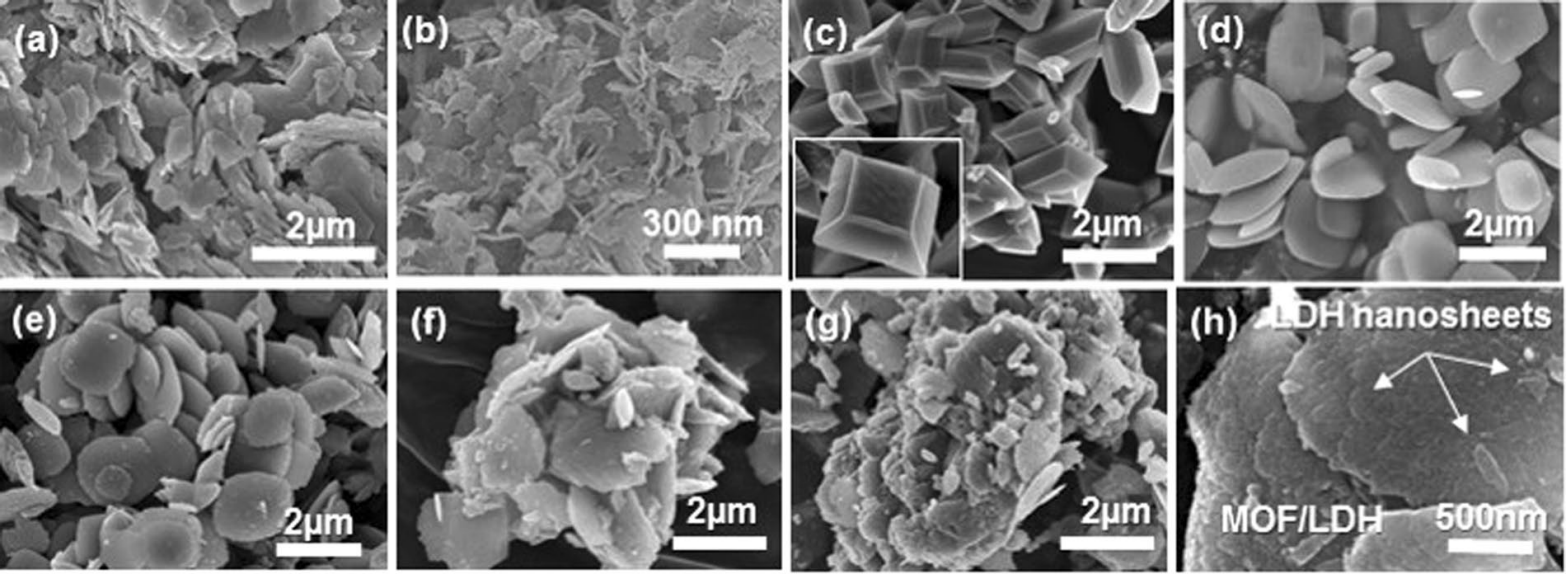
FE-SEM images of (**a**) pristine ZnCr-LDH; (**b**) exfoliated ZnCr-LDH NSs obtained by using a high-speed centrifuge; (**c**) pristine NH_2_-MIL-125(Ti); and hybrid composites: (**d**) ML50, (**e**) ML100, (**f**) ML200, and (**g**) ML400; (**h**) An enlarged image of the ML200 sample.

To elucidate the influence of ZnCr-LDH NSs on the microscopic structure of NH_2_-MIL-125(Ti) during its crystal growth, we monitored the morphology of the hybrid composites with FE-SEM. In Fig. 3a,b, as mentioned, pristine ZnCr-LDH exhibits a plate-like morphology of submicron size, while the exfoliated ZnCr-LDH NSs obtained by precipitation using a high-speed centrifuge present a randomly stacked structure consisting of sheets several hundred nanometres in size. In the case of pristine NH_2_-MIL-125(Ti) prepared in the absence of ZnCr-LDH NSs, the SEM image indicates a very regular decahedron morphology of submicron size, as shown in Fig. 3c. In contrast, the morphology of all the hybrid composites consist of a round disk-type shape, and such a disk-shape becomes more irregular with increasing amounts of ZnCr-LDH NSs (Fig. 3d–g). For closer inspection, the enlarged image of the ML200 sample shown in Fig. 3h reveals that NH_2_-MIL-125(Ti) is enwrapped by ZnCr-LDH NSs as a core-shell structure. This formation is similar to the previous result relating to reduced graphene oxide/MIL-125(Ti) composites^22^. Evidently, as the amount of ZnCr-LDH NSs increases, the crystal size and the crystallinity of NH_2_-MIL-125(Ti) becomes substantially smaller and poorer, respectively. This result agrees with that provided by the powder XRD data.

**Figure 4:**
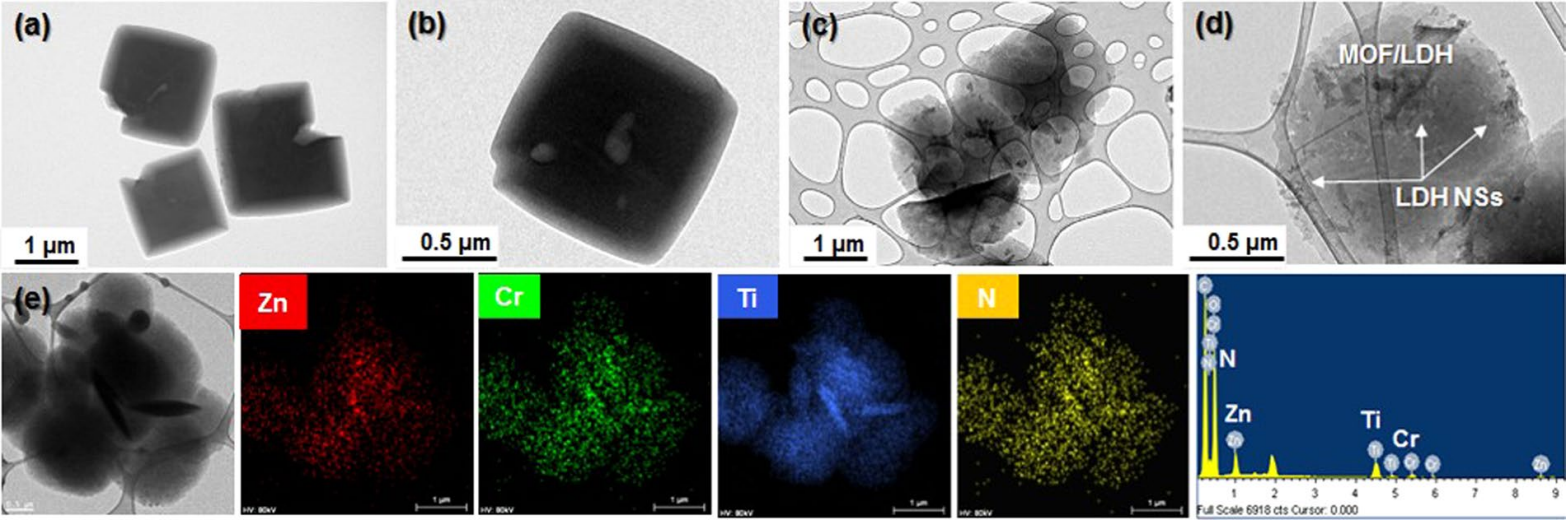
TEM images of (**a**,**b**) pristine NH_2_-MIL-125(Ti) and (**c**,**d**) the ML200 sample. (**e**) Elementary maps and the EDX spectrum of the ML200 sample.

Further, to more clearly identify the hybrid-structural formation of NH_2_-MIL-125(Ti) and ZnCr-LDH NSs, TEM images were taken before and after the hybridization. Figure 4 shows TEM images of the pristine NH_2_-MIL-125(Ti) and ML 200 samples. As with the SEM image shown in Fig. 3c, the TEM image of pristine NH_2_-MIL-125(Ti) exhibits a very regular decahedron morphology (Fig. 4a,b). After hybridization, however, images of the hybrid composite show that NH_2_-MIL-125(Ti) is enwrapped by a large amount of ZnCr-LDH NSs of several hundred nanometres in size (Figs 4c,d and S4a). In addition, HRTEM was employed to try to observe the formation of the hybrid structure. Although the lattice fringes are clearly observed (Supplementary Information, Fig. S5b), there is a severe structural distortion caused by the electron beam. The lattice distance shown in inset of Fig. S5b is calculated as 0.192 nm, corresponding to the (012) plane of ZnCr-LDH. The finding indicates that the crystallinity of ZnCr-LDH NSs remains unchanged after hybridization. Additionally, the SAED pattern also presents severe distortion, but the existence of a mixture of spots and ring patterns is confirmed (Supplementary information, inset of Fig. S5a). It is apparent that NH_2_-MIL-125(Ti) and ZnCr-LDH NSs exist in ML200.

According to the results of powder XRD, SEM, and TEM, the formation process of the hybrid composite of NH_2_-MIL-125(Ti)/ZnCr-LDH NSs can be proposed in four stages, that is, the anchoring of negatively charged ligands on the surface of ZnCr-LDH sheets, nucleation and growth through the reaction between metal ions (Ti^4+^) and ligands, and crystallization under the present reaction conditions. The driving force behind the formation of the hybrid composite is attributed to the strong electrostatic interactions. As mentioned, ZnCr-LDH NSs have a positively charged surface (Supplementary Information, Fig. S3b), and according to the previous literature^32,33^, the zeta potential of NH_2_-MIL-125(Ti) represents a negative charge (−20 to −30 mV). Indeed, when the ligand precursor is transferred into the colloidal suspension of the exfoliated ZnCr-LDH NSs, a flocculation phenomenon occurs. This indicates the ionic combination of a carboxylic group of the ligands and ZnCr-LDH NSs. Then, the metal ions (Ti^4+^) react with the other carboxylic group of the ligands. The hydrothermal reaction conditions lead to the nucleation and crystal growth. Finally, ZnCr-LDH NSs-enwrapped MOFs are generated, as illustrated in Fig. 1a. The opposite charges of these two materials result in the strong coupling by electrostatic forces. As a result, it is certain that the ZnCr-LDH NSs act as the substrate for the crystal growth of NH_2_-MIL-125(Ti) and affect the crystal size and crystallization of NH_2_-MIL-125(Ti). Similar phenomena have also been reported for 2D material-based composites^34^.

The surface area and porosity of the hybrid composites were investigated with nitrogen adsorption-desorption isotherm analysis (Supplementary Information, Fig. S6). All of the hybrid materials exhibit a steep N_2_ adsorption in the low-pressure region of *pp*_0_^−1^ < 0.1, showing typical type I characteristics for a micropore structure^35^. Additionally, the plots present a distinct hysteresis at *pp*_0_^−1^ > 0.5, indicating the presence of mesopores^35^. The calculated specific surface area, total pore volume, micropore volume, and mesopore volume are summarized in Table 1. Pristine NH_2_-MIL-125(Ti) shows a very large surface area of 1550 m^2^g^−1^ with a high pore volume of 0.636 cm^2^g^−1^, whereas ZnCr-LDH shows a significantly smaller surface area of 13 m^2^g^−1^. For the hybrid composites, all samples possess a larger surface area than that of ZnCr-LDH, whereas the value is markedly reduced as the amount of ZnCr-LDH NSs increases. It is important to note that the decrease in surface area has a negative effect on the efficiency of catalytic reactions. As is known for catalytic reactions, the surface area is an important factor that can improve the catalytic efficiency because a larger surface area can provide more surface-active sites. With this in mind, we first performed the study on the photocatalytic hydrogen production of the present hybrid composites under visible-light irradiation.

**Table 1:** Parameters taken from the nitrogen adsorption-desorption measurements.

Samples	BET surface area (m^2^/g)	Total pore volume (cm^3^/g)	^a^Micropore volume (cm^3^/g)	^b^Mesopore volume (cm^3^/g)
NH_2_-MIL-125(Ti)	1550	0.6359	0.6077	0.0282
ZnCr-LDH	13	—	—	—
ML50	911	0.4520	0.3534	0.0986
ML100	615	0.3075	0.2453	0.0622
ML200	513	0.2258	0.1850	0.0408
ML400	320	0.1902	0.1493	0.0407

^a^Determined by t-plot analysis.

^b^Mesopore volume = total pore volume − micropore volume.

**Figure 5:**
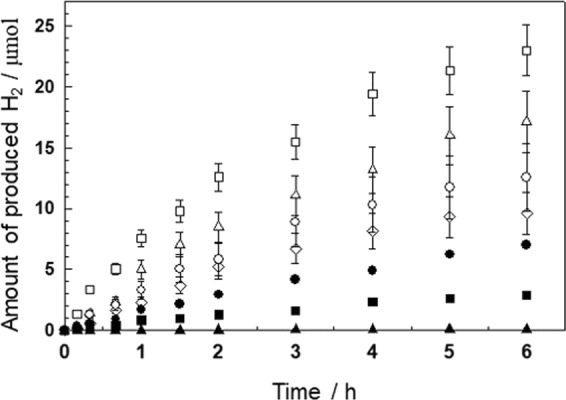
Photocatalytic hydrogen evolution over the hybrid composites ML50 (○), ML100 (Δ), ML200 (□), and ML400 (◇); pristine NH_2_-MIL-125(Ti) (●) and ZnCr-LDH (▲); and a physically mixed sample of NH_2_-MIL-125(Ti) and ZnCr-LDH NSs (■) in aqueous solutions containing 0.01 M triethanolamine (TEOA), as a hole scavenger, and under visible-light irradiation. Experimental conditions; photocatalyst: 30 mg, light source: 300 W Xe lamp with a cut-off filter (λ > 420 nm). The error bars were obtained by three measurements.

Figure 5 shows the average results of the three repeating photocatalytic tests. To our surprise, all the hybrid materials show a significantly enhanced photocatalytic performance for hydrogen production under visible-light irradiation, compared to that of pristine NH_2_-MIL-125(Ti) and ZnCr-LDH. Among the hybrid composites, in particular, the ML200 sample shows the best catalytic performance for hydrogen production. From the results of the hydrogen production test, it is important to mention that hybridization with ZnCr-LDH NSs has the excellent effect of compensating for the disadvantages, despite the surface areas of the hybrid composites being lower than that of pristine NH_2_-MIL-125(Ti). On the other hand, the photocatalytic performance of ML400, with a large amount ZnCr-LDH NSs, remarkably decreased. Such a deterioration in photocatalytic performance is attributable to the following facts: to completely exfoliate an excess of ZnCr-LDH to nanosheets is difficult under the present conditions. As seen from the results of powder XRD (Fig. 2) and SEM (Fig. 3), an excessive amount of ZnCr-LDH significantly reduces the crystallinity of NH_2_-MIL-125(Ti). Additionally, unexfoliated particles make the hybridization between the two components inhomogeneous. For those reasons, the BET surface area and total pore volume of ML400 are markedly lower, as listed in Table 1. In addition, a dense packing of excessive ZnCr-LDH on the surface of NH_2_-MIL-125(Ti) can reduce the active sites of the NH_2_-MIL-125(Ti) photocatalyst. Consequently, the reduced photocatalytic performance of ML400 is the result of an ineffective hybridization of the two components.

More importantly, in this test, the present hybrid composites show high photocatalytic activities in the absence of noble metals, such as Pt, as a co-catalyst. According to the literature, the photocatalytic activity of NH_2_-MIL-125(Ti) for hydrogen production can be improved by approximately two times when Pt is used as a co-catalyst^17^. In the present work, the average hydrogen production rate of the ML200 hybrid composite is 127.6 mmol h^−1^ g^−1^ under visible-light irradiation, which is approximately 3-times higher than that of pristine NH_2_-MIL-125(Ti) (40.8 mmol h^−1^ g^−1^) and approximately 250-times higher than that of pristine ZnCr-LDH (0.5 mmol h^−1^ g^−1^). The present values are approximately 1.5–2-times lower than the results reported for the Pt co-catalyst/NH_2_-MIL-125(Ti) system, but it is noteworthy that the photocatalytic activities of the hybrid composites are achieved without using a precious co-catalyst, such as Pt^17^. The present results are believed to benefit from the synergy of the strong coupling between NH_2_-MIL-125(Ti) and ZnCr-LDH NSs. Additionally, to confirm this notion, a physically mixed sample of NH_2_-MIL-125(Ti) and ZnCr-LDH NSs was prepared by adding NH_2_-MIL-125(Ti) powder to the colloidal suspension of ZnCr-LDH NSs, and the photocatalytic activity was tested. As shown in Fig. 5, this sample exhibits a photocatalytic activity (16.1 mmol h^−1^ g^−1^) even lower than that of pristine NH_2_-MIL-125(Ti). This finding verifies that there exists a strong chemical interaction between NH_2_-MIL-125(Ti) and ZnCr-LDH NSs in the hybrid composites. Based on the photocatalytic performances, further investigations were conducted for the ML200 sample.

To investigate the coexistence of NH_2_-MIL-125(Ti) and ZnCr-LDH NSs, elementary mapping and energy-dispersive X-ray spectroscopy (EDX) were conducted (Fig. 4e). Elementary mapping images and the EDX spectrum show that each transition metal element of Zn, Cr, and Ti, together with N, exists and is distributed homogenously in the ML200 sample. The molar ratio of Ti: Zn: Cr is 1: 0.75: 0.34, and therefore, the molar ratio of {Ti_8_O_8_(OH)_4_(BDC-NH_2_)_6_}/(Zn_1−x_Cr_x_(OH)_2_) is determined to be 0.17. As mentioned previously, the existence of ZnCr-LDH NSs is not verified by the powder XRD patterns. Therefore, to cross-confirm the HRTEM image (Supplementary Information, Fig. S5b), Zn K- and Cr K-edge X-ray absorption near-edge spectroscopy (XANES) of the ML200 sample was performed (Supplementary Information, Fig. S7). The Zn K- and Cr K-edge XANES spectral shapes of the ML200 hybrid composite are quite similar to those of pristine ZnCr-LDH, indicating that the structure of ZnCr-LDH NSs remains intact after the hybridization. This is in good agreement with the result obtained from the HRTEM image.

**Figure 6:**
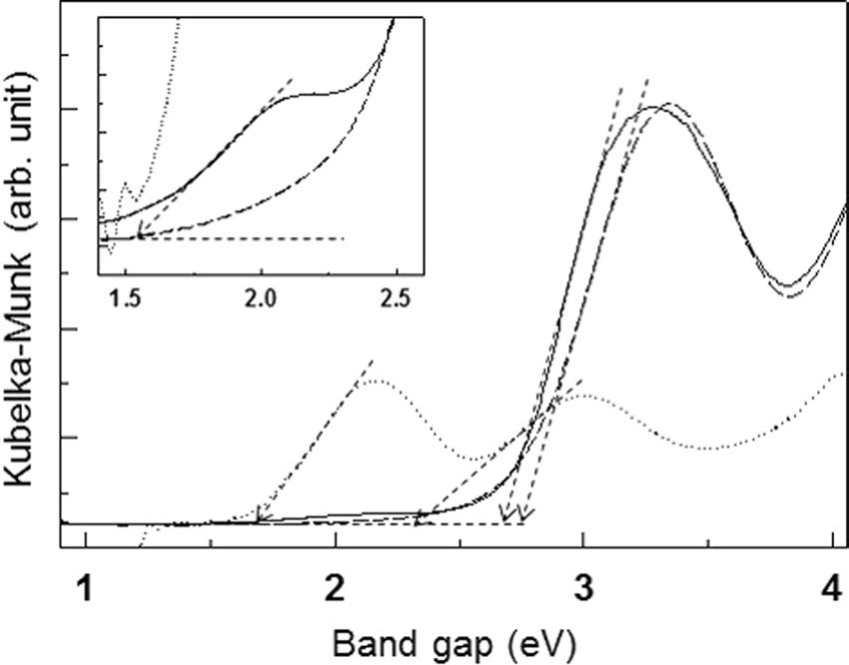
UV-vis diffuse reflectance spectra of the ML200 hybrid composite (solid line) and unhybridized samples of NH_2_-MIL-125(Ti) (dashed line) and ZnCr-LDH (dotted line).

UV-vis diffuse reflectance spectra (DRS) were obtained to characterize the band structure and optical properties of the ML200 hybrid composite in comparison with those of each compound before hybridization. As plotted in Fig. 6, pristine NH_2_-MIL-125 possesses one strong absorption peak at 2.76 eV corresponding to the ligand-to-metal-charge transfer (LMCT) of the valence band (VB) composed of C 2p, N 2p, and O 2p orbitals → the conduction band (CB) composed of Ti 3d and O 2p orbitals^18,36^. In the case of ZnCr-LDH, two strong absorption peaks at 1.71 eV for Cr 3d_t2g_ → Cr 3 d_eg_ (transition) and at 2.37 eV for the ligand-to-metal-charge transfer (LMCT) of O 2p → Cr 3 d_eg_ are observed^27,37^. Upon the hybridization of the two materials, the ML200 hybrid composite displays two absorption peaks at 1.54 nm and 2.69 eV in the visible region. The spectral difference between before and after the hybridization is attributed to the effective electronic coupling in the hybridization of the two materials, that is, an overlap occurs between the d-d transition of chromium ions in ZnCr-LDH and that of titanium ions in NH_2_-MIL-125(Ti).

**Figure 7:**
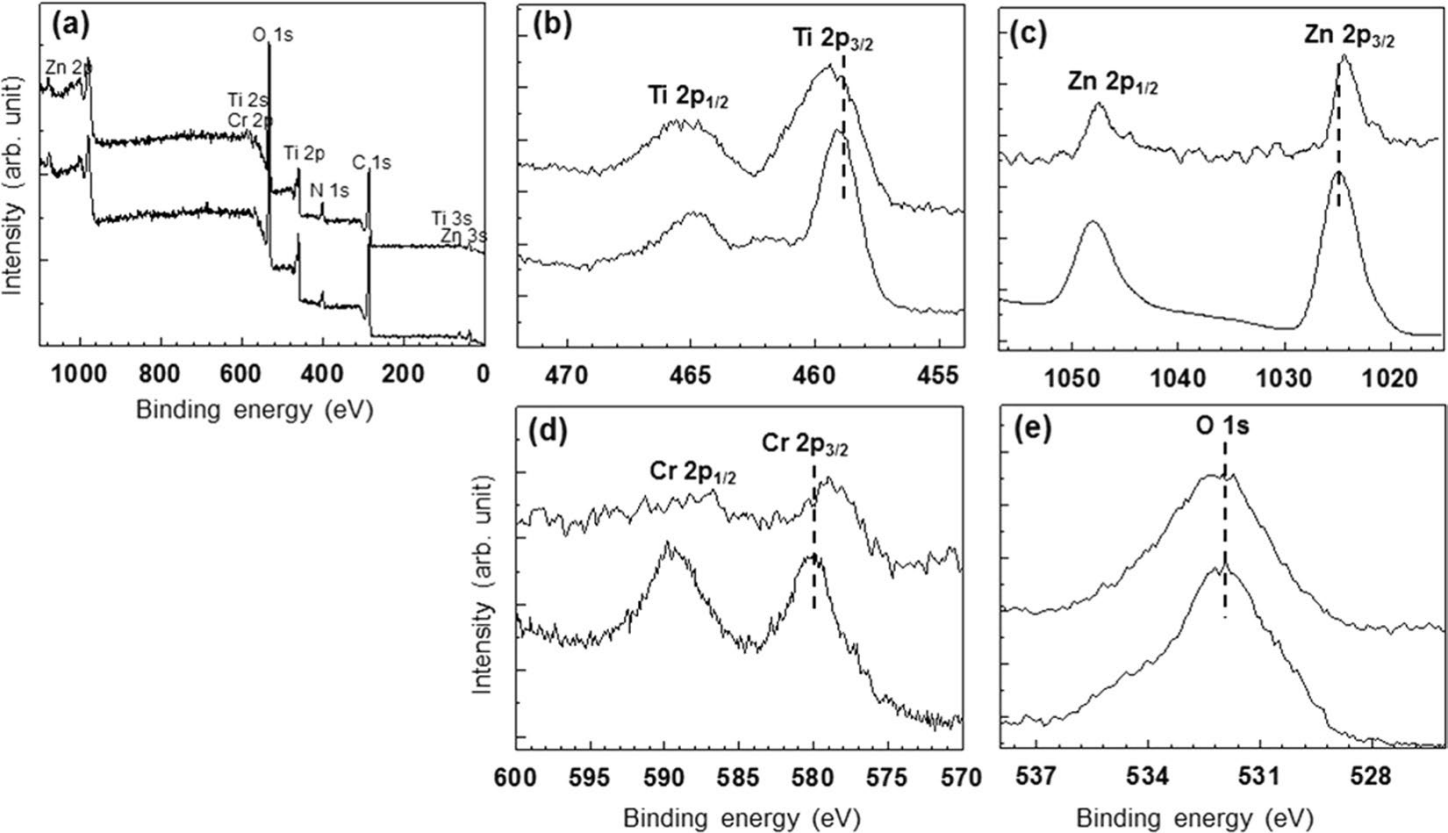
XPS spectra of (bottom in **a**,**b**,**e**) pristine NH_2_-MIL-125(Ti), (bottom in **c**,**d**) pristine ZnCr-LDH and (top in **a**–**e**) the ML200 hybrid composite: (**a**) the full-spectrum survey, (**b**) Ti 2p, (**c**) Zn 2p, (**d**) Cr 2p and (**e**) O 1 s.

X-ray photoelectron spectroscopy (XPS) was carried out for the ML200 sample. As shown in Fig. 7b, the Ti 2p binding energy of the ML200 hybrid composite is shifted to a higher binding energy after the hybridization with ZnCr-LDH NSs. On the other hand, the binding energies of the Zn 2p and Cr 2p are negatively shifted, as shown in Fig. 7c,d. Further, in the case of the XPS spectrum of the O 1 s, the overall peak is slightly shifted to a higher binding energy after hybridization (Fig. 7e). Such changes in the binding energies are generated because the electron density of NH_2_-MIL-125(Ti) decreases and that of ZnCr-LDH NSs increases. These findings clearly indicate that there exists an effective electronic coupling between the two components because of the hybridization, leading to not only the harvest of more incident photons to produce more photo-generated electrons and holes but also the suppression of the recombination of photo-generated electron and hole pairs due to an effective electron transfer between NH_2_-MIL-125(Ti) and ZnCr-LDH NSs^38,39^.

In addition, FT-IR spectroscopy was carried out to prove the hybridization of ZnCr-LDH NSs and NH_2_-MIL-125(Ti) (Supplementary Information, Fig. S8). In the case of pristine ZnCr-LDH, strong and broad peaks centred at 3300–3600 cm^−1^ and 1635 cm^−1^ are observed and are assigned as the O-H stretching vibration of the hydroxyl groups in the brucite layers and the H-O-H bending vibration of interlayer water molecules, respectively^40^. In addition, a characteristic of ZnCr-LDH in its nitrate form is exhibited as an absorption peak around 1340 cm^−1^ for the interlayer NO_3_^−^ vibration band. The bands observed below 700 cm^−1^ represent Zn/Cr–OH and Zn–O–Cr stretching vibration modes^40^. Pristine NH_2_-MIL-125(Ti) and the ML200 hybrid composite exhibit similar characteristic bands: the carboxylic acid functional groups of NH_2_-MIL-125(Ti) in the region of 1380–1700 cm^−1^ and the vibrations of the O-Ti-O groups in the region of 400–800 cm^−1^ ^41^. The ML200 hybrid composite shows all the characteristic peaks of ZnCr-LDH and NH_2_-MIL-125(Ti) around 3300–3600 cm^−1^, clearly confirming the presence of the two components. Especially, a strong band at 635 cm^−1^ corresponding to O-Ti-O is negatively shifted after the hybridization. This change is due to the decrease in the electron density of NH_2_-MIL-125(Ti), which is in good agreement with XPS results (Fig. 7). The present FTIR spectroscopy analysis not only strongly confirms the existence of the ZnCr-LDH and NH_2_-MIL-125(Ti) but also indicates that there is an apparent electronic interaction between the two materials.

The effective electronic interaction between the two materials was investigated by the photocurrent transient response measurement (Supplementary Information, Fig. S9). The photocurrent response under on-off light operation demonstrates that the photocurrent of the ML200 hybrid composite is considerably higher than that of pristine NH_2_-MIL-125(Ti) and ZnCr-LDH electrodes. This finding is attributed to the effective electron coupling brought about by the hybridization of ZnCr-LDH and NH_2_-MIL-125(Ti). Consequently, the efficiency of the separation and transfer of photogenerated electron–hole pairs is significantly improved.

## Discussion

**Figure 8:**
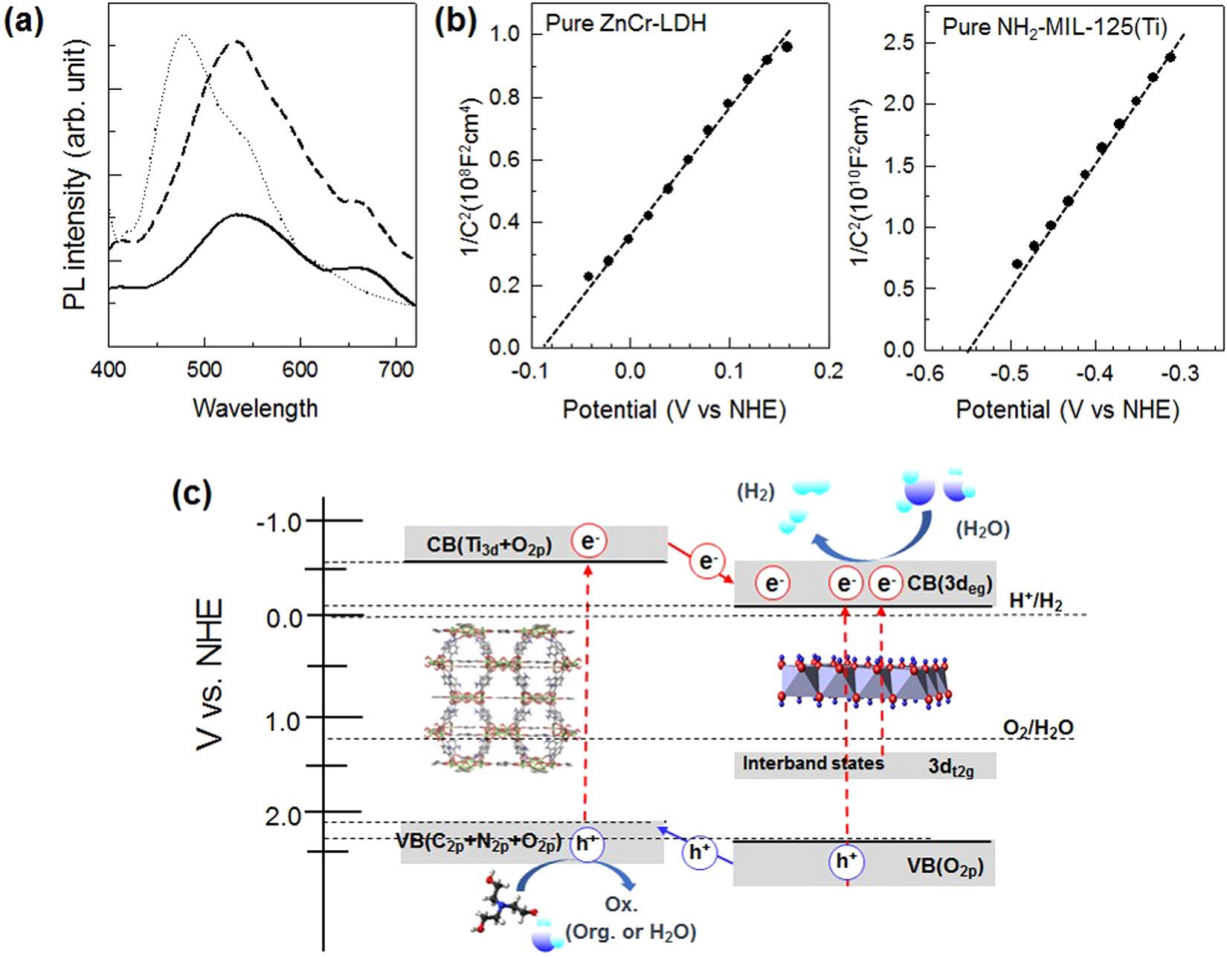
(**a**) PL spectrum of the ML200 hybrid composite (solid line), together with that of pristine NH_2_-MIL-125(Ti) (dashed line) and ZnCr-LDH (dotted line). (**b**) Mott-Schottky plots of pure ZnCr-LDH and pure NH_2_-MIL-125(Ti). (**c**) An illustration of the proposed photocatalytic mechanism derived from the results obtained in this work.

The enhancement in the photocatalytic activity of the hybrid composite was elucidated by performing photoluminescence spectroscopy (PL) and the electrochemical analysis. In Fig. 8a, pristine NH_2_-MIL-125(Ti) and ZnCr-LDH show a strong PL peak around 538 nm and 476 nm, respectively. For ML200, the PL peak intensity weakens and broadens significantly, suggesting the effective electron transfer of the photo-generated electron and hole between NH_2_-MIL-125(Ti) and ZnCr-LDH NSs. Such an electron transfer can be supported by the results obtained from the Mott-Schottky plots. As plotted in Fig. 8b, both ZnCr-LDH and NH_2_-MIL-125(Ti) show a positive slop in the MS plots, indicating n-type semiconductor characteristics. The flat-band potentials of NH_2_-MIL-125(Ti) and ZnCr-LDH are −0.55 V and −0.09 V vs NHE, respectively. Therefore, combined with the results obtained from the UV-vis spectra, the highest valence band edges are determined to be 2.21 eV for the NH_2_-MIL-125(Ti) and 2.28 eV for ZnCr-LDH. These results are similar to those of previous reports^30,42–44^.

From the results of the Mott-Schottky plots and UV-Vis spectroscopy, a possible mechanism of the photocatalytic process of the hybrid composites under visible-light irradiation is proposed and illustrated in Fig. 8c. Two catalysts absorb incident photons and generate electrons and holes, which are separated. The photo-exited electrons in the CB of the NH_2_-MIL-125(Ti) catalyst can move to the CB of ZnCr-LDH (or reductive surface sites), whereas the holes can move to the VB (or oxidative surface sites) of NH_2_-MIL-125(Ti) from that of ZnCr-LDH^38^. Photo-generated electrons in CB of ZnCr-LDH are finally used to reduce water molecules, resulting in efficient hydrogen production^38^. Evidently, the hybridization of these two materials provides a more efficient spatial separation of electron-hole pairs than does each material individually and consequently enhances the photocatalytic activity. In addition, as seen from Table 1, the total surface area and micropore volume are reduced for the hybrid composites, but hybridization with ZnCr-LDH NSs increases the mesopore volume. This mesoporosity is attributed to the formation of voided spaces between NH_2_-MIL-125(Ti) and ZnCr-LDH NSs. This result is consistent with the SEM and TEM results. Considering the smooth approach of water molecules to the pores, the increase in mesopores increases the effective contact between the surface and the water molecules compared to the micropore-only case. Therefore, the mesopores help improve the photocatalytic reactivity.

**Figure 9:**
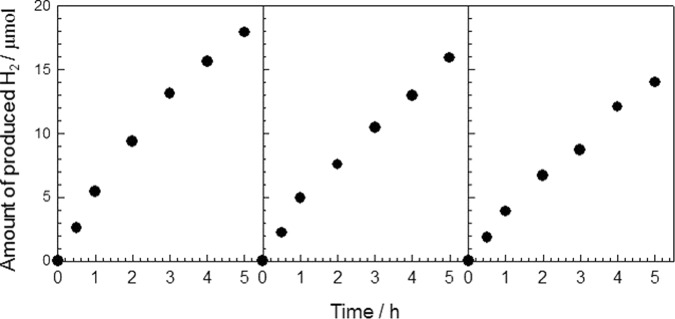
Recycling test of the ML200 sample for photocatalytic hydrogen production under visible-light irradiation (λ > 420 nm). The photocatalytic experiment was performed for 15 h, with evacuation every 5 h.

The recycling test of hydrogen production was carried out to explore the durability of the hybrid composite. As plotted in Fig. 9. the photocatalytic performance over the ML200 sample remains constant with a slight decrease for three consecutive photocatalytic reactions, showing the photo-chemical stability of the present hybrid composites. To find the origin of the decrease in photocatalytic activity, the powder XRD and FE-SEM of the recovered hybrid composite were performed (Supplementary Information, Fig. S10). The results clearly demonstrate the maintenance of the hybrid’s original structure and morphology after the recycling test, underscoring the high durability of this composite. One possibility for the photocatalytic activity decrease would be the consumption of a sacrificial agent. To probe such a possibility, the recovered hybrid composite was retested for the photocatalytic reaction with the addition of a sacrificial agent. This experiment confirmed that the photocatalytic hydrogen production can be restored (Supplementary Information, Fig. S11).

In addition to solar fuel production issues, studies on environmental remediation by using photocatalysts have been actively proceeding. Therefore, the present ML200 hybrid composite was applied to the photocatalytic decomposition of an organic dye (i.e., methylene blue) under visible-light irradiation (Supplementary Information, Fig. S12). As a result, the photocatalytic activity of the ML200 hybrid composite is superior to that of ZnCr-LDH, with a high oxidizing power, as well as that of pristine NH_2_-MIL-125(Ti). This finding also shows the potential of the present hybrid composites for expansion into different photocatalyst applications.

In the present study, we developed a novel visible-light-active photocatalyst for hydrogen production based on NH_2_-MIL-125(Ti) and ZnCr-LDH NSs. The hybridization of NH_2_-MIL-125(Ti) with ZnCr-LDH NSs leads to a significant change in the morphology and the optical properties of NH_2_-MIL-125(Ti). Compared to pristine NH_2_-MIL-125(Ti), with a high surface area, the hybrid composites exhibit enhanced photocatalytic activities and excellent photo-chemical stability for hydrogen production under visible-light irradiation, despite their low surface areas. Such enhanced photocatalytic activities can be ascribed to the strong electronic coupling between the two components, the expanded light absorption, and the band alignment to enhance the lifetime of photoinduced electrons and holes. In addition, these hybrid composite photocatalysts can also be applied for environment remediation. These results demonstrate that the present synthesis approach can develop various new photocatalysts based on MOFs.

## Methods

### Preparation of hybrid composites of NH_2_-MIL-125(Ti) and ZnCr-LDH nanosheets

First, pristine ZnCr-LDH in the nitrate form was prepared by the co-precipitation method, similar to the reported procedure^30^. First, a mixture of Zn(NO_3_)_2_∙6H_2_O, Cr(NO_3_)_3_∙9H_2_O and NaNO_3_ with a Zn/Cr/NO_3_ molar ratio of 2: 1: 3, was dissolved in 100 mL of deionized water. The water was decarbonated by boiling under an N_2_ atmosphere before using. The mixed solution was titrated with 1.0 M NaOH up to pH 10.5 with vigorous stirring at room temperature. Then, the reaction was carried out at 60 °C for 24 h under an N_2_ atmosphere. After the reaction, a bright-purple slurry was separated by centrifugation, then washed with decarbonated water to thoroughly remove unreacted ions, and finally, vacuum-dried at 60 °C overnight. The colloidal suspension of exfoliated ZnCr-LDH nanosheets was obtained by alternatively vigorously stirring and ultrasonicating the LDH powdery sample in a mixed solvent (1:1) of dimethylformamide (DMF) and methyl alcohol (MeOH). In this work, to find the optimum photocatalytic property of the hybrid composite catalysts, the amount of ZnCr-LDH was adjusted to 50, 100, 200, and 400 mg when preparing a colloidal suspension of ZnCr-LDH nanosheets. For the case when 400 mg of ZnCr-LDH was used, a large portion of ZnCr-LDH was not able to be exfoliated. The hybrid composites of NH_2_-MIL-125(Ti) and ZnCr-LDH were prepared by growing NH_2_-MIL-125(Ti) in the colloidal suspension of ZnCr-LDH nanosheets. BDC-NH_2_ (2-aminoterephthalic acid, 1.08 g) was dissolved in an anhydrous DMF/MeOH (1:1) mixed solution containing exfoliated ZnCr-LDH nanosheets and treated by a sonicator. Then, Ti(BuO)_4_ (titanium butoxide, 0.51 mL) was added to the solution, which was stirred. The mixture was solvothermally treated in a Teflon-lined stainless autoclave at 150 °C for 16 h, followed by separation by using centrifugation, then washing with DMF and MeOH, and finally drying at 60 °C overnight. For comparison, pure NH_2_-MIL-125(Ti) was prepared via the same procedure above but without ZnCr-LDH nanosheets.

### Characterization

The crystal structures of the resultant hybrid composites were characterized using powder X-ray diffraction (powder XRD, Rigaku, Cu kα = 1.5418 Å, 298 K) measurements. Their crystallite morphologies were probed using scanning electron microscopy (SEM, Hitachi S3400-N) and transmission electron microscopy (TEM) images (JEOL JEM-2100, 200 kV). To obtain the chemical composition of the hybrid composites, a Hitachi S3400-N microscope equipped with an energy dispersive X-ray spectrometer (EDX, Horiba EMAX) was employed. UV-vis diffuse reflectance spectra were recorded on a Shimadzu UV-2550 spectrophotometer equipped with an integrating sphere using BaSO_4_ as the reference. The nitrogen adsorption–desorption isotherms (a Nova 1200e instrument) for the specific surface area and porosity were measured volumetrically at 77 K using ultrapure nitrogen gas and in which all samples were degassed at 150 °C for 2 h. The XANES data were collected at the Ti K-edge on beamline 10 C at the Pohang Accelerator Laboratory (PAL) in Pohang, Korea. The XAS measurements were carried out at room temperature in transmission mode using gas-ionization detectors. All the present spectra were calibrated by measuring the spectrum of Ti metal foil. X-ray photoelectron spectroscopy (XPS) measurements were obtained with a MultiLab 2000 spectrometer equipped with a micro-focusing monochromated Al Kα X-ray (1486.6 eV) source. The photoluminescence (PL) spectra were obtained using a LabRAM HR-800 spectrophotometer with an excitation wavelength of 325 nm. Fourier-transform infrared (FT-IR) spectra were collected to confirm the functional groups of the samples. The KBr pellet technique was used for the FT-IR measurement. The photocurrent-response measurements were carried out with a SP-240 potentiostat/EIS (BioLogic Science Instrument) using a conventional three-electrode cell. The three-electrode setup was used with 0.5 M Na_2_SO_4_ (pH 6.5). Ag/AgCl (3 M KCl, 0.210 V vs. NHE) and Pt mesh were used as the reference electrode and counter electrode, respectively. The working electrodes were prepared by pasting a uniform layer of the sample on fluorine-doped tin oxide (FTO) glass with an active area of ca. 0.2 cm^2^. The supporting electrolyte was a 0.5 M Na_2_SO_4_ aqueous solution, and a visible-light irradiation system comprised of a 300 W Xe lamp, as a light source, was used. To find the flat-band potential, Mott-Schottky plots were obtained for the pure NH_2_-MIL-125(Ti) and ZnCr-LDH using a SP-240 potentiostat/EIS (BioLogic Science Instrument) under the three-electrode cell conditions, as previously mentioned. A sinusoidal modulation of 10 mV was applied at a frequency of 1 kHz.

### Photocatalytic reactions

#### Photocatalytic test for hydrogen production

The photocatalytic reactions for hydrogen production were conducted in a Pyrex reactor connected to a closed-gas circulation system made of glass. The photoreduction of H_2_O to H_2_ was performed in an aqueous solution at room temperature under visible-light irradiation using the light system mentioned in the dye degradation test. The catalyst (30 mg) was dispersed by stirring in a 50 mL aqueous solution containing 0.01 M triethanolamine (TEOA) as a sacrificial reagent. Prior to irradiation, the airtight reactor was thoroughly degassed to remove air. A gentle magnetic stirrer was applied at the bottom of the reactor. The test for photocatalytic hydrogen production was evaluated under visible-light irradiation (a light system comprised of a 300 W Xe lamp as a light source, a water filter to remove the IR region, and a cut-off filter to achieve visible light (λ > 420 nm). The amount of hydrogen produced was determined by taking 100 μL of gas from the headspace of the reactor using a syringe and injecting it into the gas-sampling loop of a gas chromatograph (Shimadzu GC7890II, TCD detector, molecular sieve columns and N_2_ carrier) every hour.

#### Decomposition test of methylene blue (MB) dye

The hybrid composite (30 mg) was dispersed in a solution of methylene blue dye, prepared by dissolving MB dye (50 μM) in distilled water. To achieve the adsorption-desorption equilibrium state between dye and sample, the solution was vigorously stirred in the dark for 1 h. The experimental conditions for photocatalytic dye decomposition were similar to those for the hydrogen production test. Every hour, 5 mL sample was taken from the photocatalytic reactor using a syringe and filtered by a 0.45 μm polytetrafluoroethelene filter to remove the catalyst sample. The degradation of MB dye was monitored by measuring the absorbance at λ = 664 nm as a function of irradiation time with a UV-vis spectrophotometer.

## Supplementary information

Supplementary Info
