# Supplementary material for: Enhanced photocatalytic performance of a Ti-based metal-organic framework for hydrogen production: Hybridization with ZnCr-LDH nanosheets
Source: Sci Rep. 2019 May 20;9:7584. doi: 10.1038/s41598-019-44008-6 (PMC6527569; doi:10.1038/s41598-019-44008-6)
Supplement: Supplementary file 1 — Supplementary Info [file 41598_2019_44008_MOESM1_ESM.docx]

**Supplementary Information**

**Enhanced photocatalytic performance of a Ti-based metal-organic framework for hydrogen production: Hybridization with ZnCr-LDH nanosheets**

Muhammad Sohail^1,2,†^, Hyunuk Kim^1^ & Tae Woo Kim^1, †,^*

^1^Energy Materials Laboratory, Korea Institute of Energy Research, 152 Gajeong-ro, Yuseong-gu, Daejeon 34129, Republic of Korea

^2^Advanced Energy Technology, University of Science and Technology, 217 Gajeong-ro, Yuseong-gu, Daejeon 34113, Republic of Korea

^†^M.S. and T.W.K. contributed equally to this work.

*Corresponding author.

: Tae Woo Kim (E-mail: twkim2015@kier.re.kr)

**
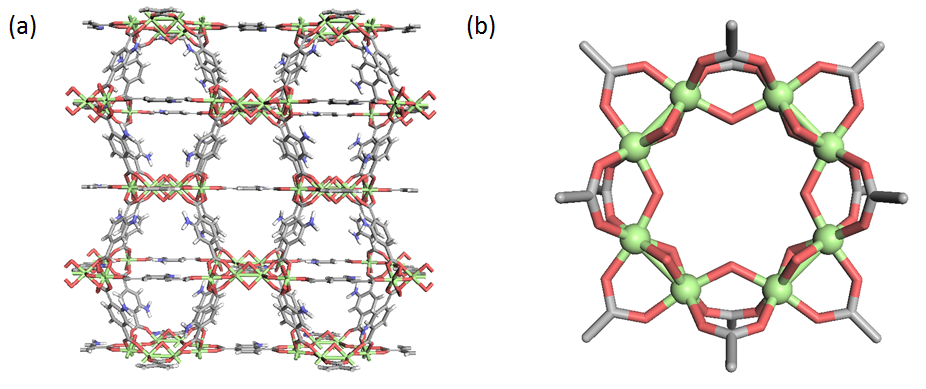
**

**Fig. S1.** (a) The crystal structure of NH_2_-MIL-125(Ti) and (b) the enlarged skeleton of Ti cluster.


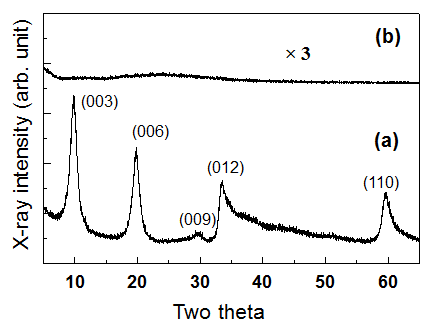


**Fig. S2.** PXRD patterns of (a) pristine ZnCr-LDH synthesized by co-precipitation and (b) exfoliated ZnCr-LDH nanosheets.

**
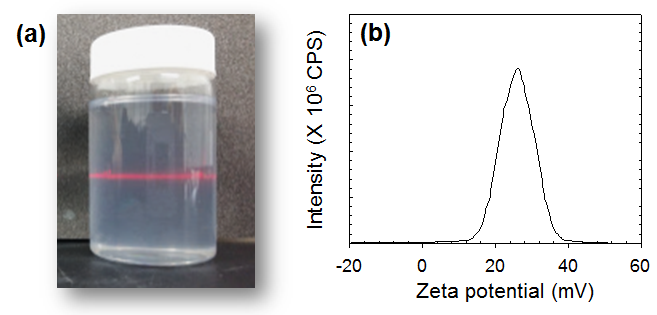
**

**Fig. S3**. (a) Tyndall effect and (b) Zeta potential of the exfoliated ZnCr-LDH nanosheets.

**
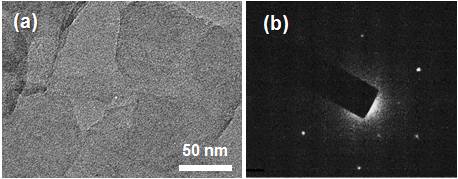
**

**Fig. S4**. (a) TEM image and (b) SAED pattern of the exfoliated ZnCr-LDH nanosheets.


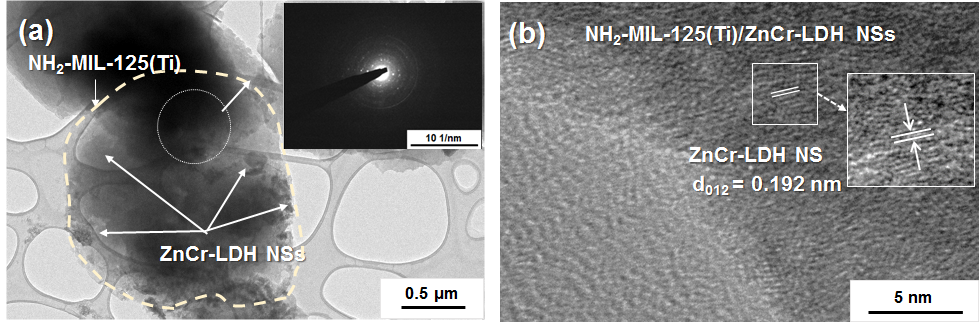


**Fig. S5**. (a) TEM images and (b) HRTEM image for ML200 sample. The inset in (a) shows SAED pattern.

**
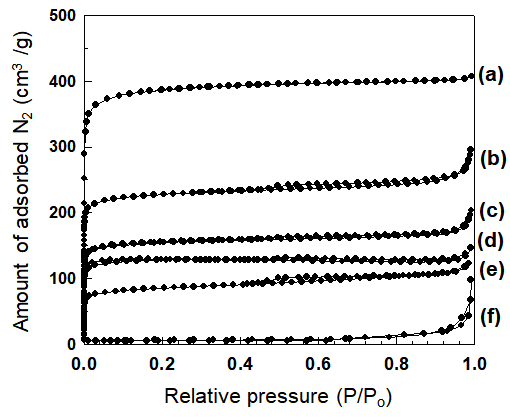
**

**Fig. S6.** Nitrogen adsorption-desorption plots; (a) NH_2_-MIL-125(Ti), (b) ML50, (c) ML100, (d) ML200, (e) ML400, and (f) ZnCr-LDH.

**
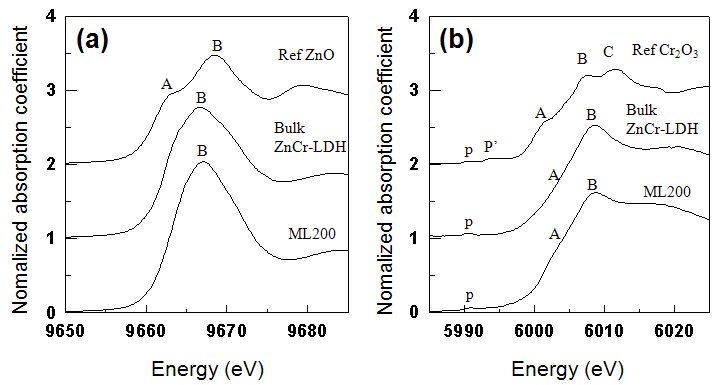
**

**Fig. S7.** (a) Zn K- and (b) Cr K-edge XANES spectra of ML200 sample and references of ZnO and Cr_2_O_3_.


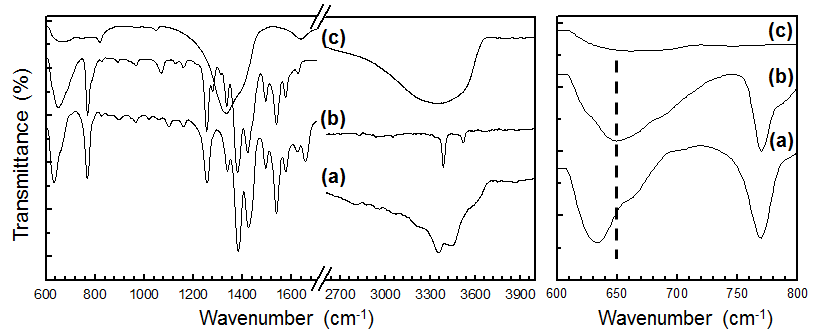


**Fig. S8.** FT-IR spectra of (a) ML200 hybrid composite, (b) pristine NH_2_-MIL-125(Ti), and (c) pristine ZnCr-LDH. Left panel shows the region in the range of 600-800 cm^-1^

**
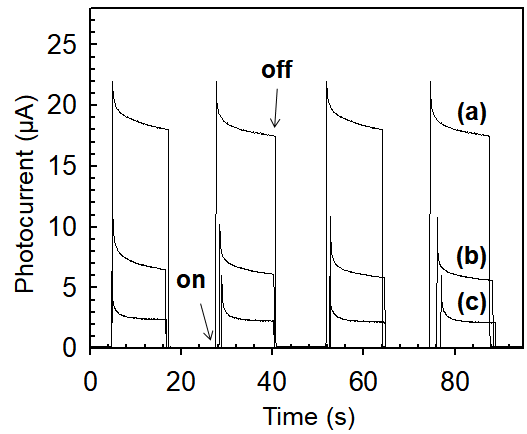
**

**Fig. S9.** The photocurrent transient response measurement of (a) ML200 hybrid composite, together with (b) pristine NH_2_-MIL-125(Ti) and (c) ZnCr-LDH.

**
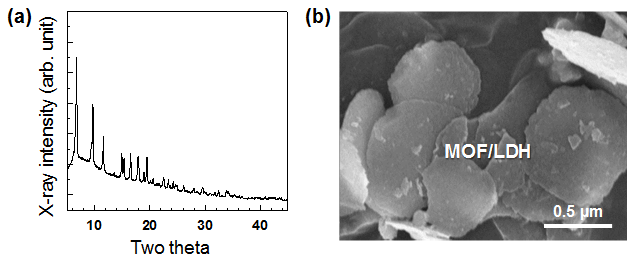
**

**Fig. S10.** XRD pattern and SEM image of the recovered ML200 hybrid composite.


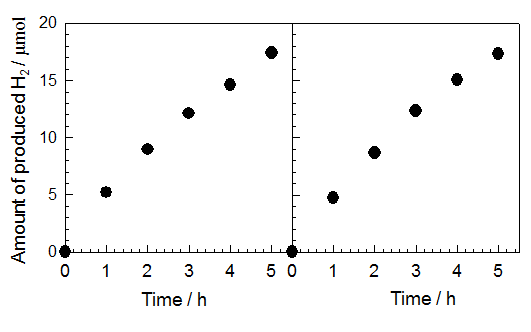


**Fig. S11**. Effect of addition of sacrificial agent on recycling of the recovered ML200 sample after photoreaction for 15h.

**
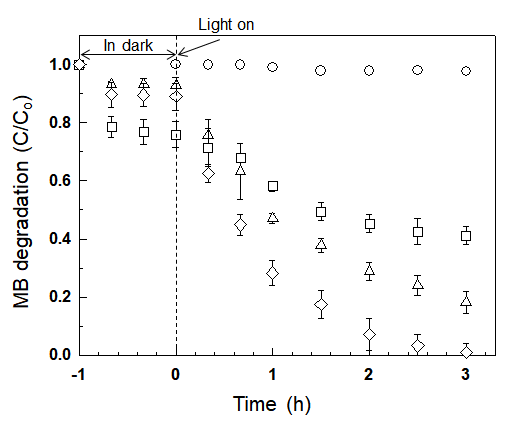
**

**Fig. S12.** Photocatalytic decomposition of methylene blue dye; blank (open circles), pristine ZnCr-LDH (open triangles), NH_2_-MIL-125(Ti) (open squares), and ML200 sample (open diamonds). The error bars were obtained by three measurements.
